# Soft tissue pseudotumors of the hand and wrist mimicking malignancy: Two case reports

**DOI:** 10.1097/MD.0000000000034236

**Published:** 2023-06-30

**Authors:** Jong Ok Kim, Yeon Soo Lee, Sangeun Park

**Affiliations:** a Department of Pathology, Daejeon St. Mary’s Hospital, The Catholic University of Korea, Chung-gu, Daejeon, Republic of Korea; b Department of Radiology, Daejeon St. Mary’s Hospital, The Catholic University of Korea, Chung-gu, Daejeon, Republic of Korea; c Department of Orthopedic Surgery, Daejeon St. Mary’s Hospital, The Catholic University of Korea, Chung-gu, Daejeon, Republic of Korea.

**Keywords:** case reports, hand and wrist, IgG4-related disease, magnetic resonance imaging, malignancy, pseudotumor

## Abstract

Most soft-tissue lesions in the hand and wrist have a benign etiology, and malignant tumors, such as soft-tissue sarcomas, are rare. Mimickers of soft tissue tumors in the hand and wrist are more common than true neoplastic lesions; however, soft tissue pseudotumors that mimic malignancy are very rare.

**Patient concerns:**

This study describes 2 patients with soft tissue pseudotumors of the hand and wrist. Both patients presented with rapidly growing soft-tissue masses. Magnetic resonance imaging (MRI) revealed ill-defined margins and an aggressive appearance in both cases, leading to a strong suspicion of malignant soft tissue tumors.

**Diagnosis:**

Both patients underwent incisional biopsies, and the final diagnoses were inflammation due to IgG4-related disease in the first case and chronic granulomatous inflammation in the second case.

**Interventions:**

The first patient was administered oral steroids, while the second patient was treated with anti-inflammatory drugs.

**Outcomes:**

Both patients showed a reduction in swelling of the hand and wrist.

**Lessons:**

Although the imaging approach for pseudotumorous lesions is similar to that for true soft tissue tumors, the management of these lesions is different. Biopsies should only be performed when the diagnosis is unclear.

## 1. Introduction

Soft tissue masses in the hand and wrist can be categorized as pseudotumors, benign tumors, or malignant tumors. Most tumors are benign, and malignant tumors in this location are rare.^[1–4]^ However, the incidence of soft tissue pseudotumors in the hands and wrists is underestimated.^[2]^

There have been several reports of pseudotumoral soft tissue lesions of the hand and wrist.^[1–8]^ However, no cases of hand or wrist pseudotumors mimicking malignancy have been reported in the literature.

The ability of magnetic resonance imaging (MRI) to differentiate between benign and malignant soft tissue masses in the hand and wrist is controversial. MRI findings indicative of malignancy include large size (>5 cm), deep location, peritumoral edema, skin thickening, lobulation, fascial edema, hemorrhage, and necrosis.^[1,3,4]^ However, malignant tumors of the hand and wrist can also be small and superficial, and some sarcomas present as well-defined masses without peritumoral edema.^[6]^ Conversely, infection or inflammation can present as masses with ill-defined contours or perilesional or fascial edema. Therefore, histological analysis may be necessary for definitive diagnosis.

We report 2 cases of pathologically confirmed soft tissue hand and wrist pseudotumors that were mistaken for malignancies. We describe the MRI and corresponding pathologic findings and discuss the importance of histologic diagnosis in cases with ambiguous imaging results.

## 2. Case presentation

### 2.1. Case report 1

A 54-year-old woman presented with a palpable mass on the ulnar side of her left wrist that had first become apparent 3 months earlier. The mass was movable, hard, painful, and had increased in size since its appearance. The patient had no relevant medical histories.

**Figure 1.**
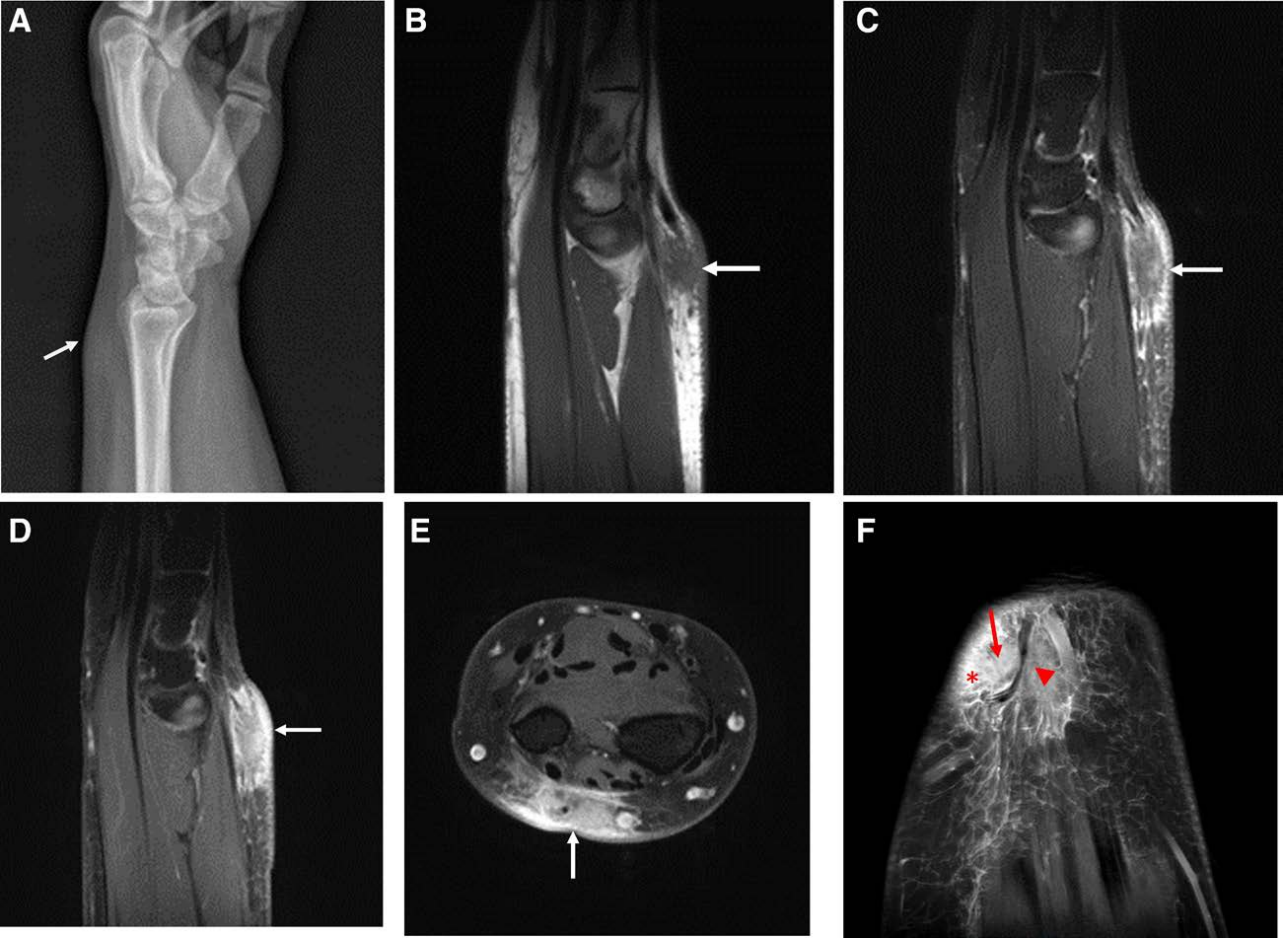
Plain lateral radiograph of the left wrist (A) shows a focal soft tissue swelling at the dorsal aspect (arrow). MRI of the wrist demonstrates an ill defined soft tissue mass (arrow) with low signal intensity (SI) on sagittal T1 weighted image (WI) (B) and intermediate SI on sagittal fat suppressed T2 WI (C). Postcontrast fat suppressed MR images (D–F) show ill defined, heterogeneously enhancement of the mass (arrows) with enhanced peritumoral edema (star in F) and neurovascular infiltrative appearance. However, vascular structures (arrowhead in F) in the enhanced mass appear intact. MRI = magnetic resonance imaging.

Plain radiography revealed focal swelling of the soft tissue in the dorsal left wrist (Fig. 1A). MRI revealed an ill-defined, 2 cm sized soft tissue mass with low signal intensity (SI) on T1-weighted images and low to intermediate SI on T2WI at the dorsal subcutaneous aspect of the distal radioulnar level (Fig. 1B–D). On postcontrast images (Fig. 1E and F), the mass showed heterogeneous enhancement and a superficial neurovascular infiltrative appearance. Enhanced subcutaneous edema around the mass and some enhancement of the adjacent superficial fascia were observed. Based on these MRI features, the differential diagnosis suggested a malignant tumor such as undifferentiated pleomorphic sarcoma (UPS), synovial sarcoma, fibrosarcoma, or epithelioid sarcoma, or a less likely possibility of a pseudotumor.

Because we could not be sufficiently certain whether the mass was benign or malignant based on imaging information, an incisional biopsy was performed. The mass was hard, palpable and rubbery. It was subcutaneously located on the dorsoulnar side of the left wrist. Histopathological examination revealed the absence of malignant cells. The mass was found to be inflamed, most likely due to immunoglobulin G4-related disease (IgG4-RD).

**Figure 2.**
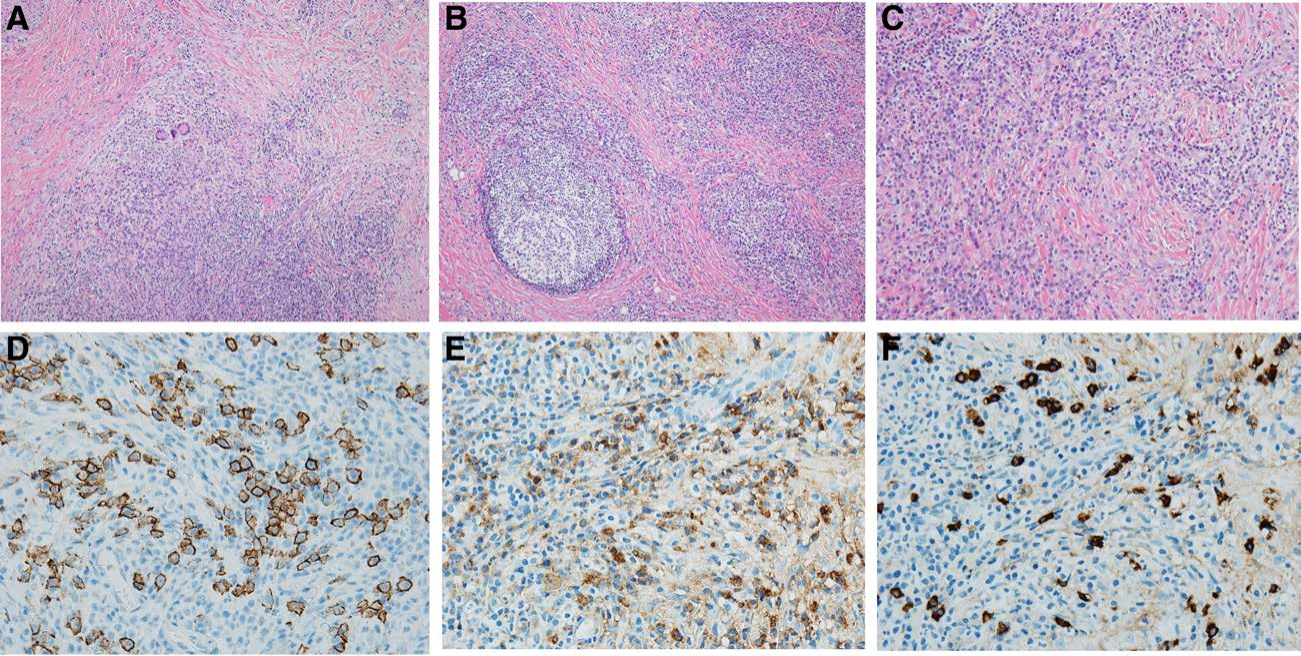
Slides show lymphoplasma cell infiltrate, foreign-body type giant cells (A), lymphoid follicle formation (B), eosinophils, and neutrophils (C) in the background of fibrosis on Hematoxylin and eosin slide (H&E X 100, A, B, C). Plasma cells are immunohistochemically positive for CD138 (D), IgG (E), and IgG4 (F) (immunohistochemical slide X 400, D, E, F).

Hematoxylin and eosin slides (Fig. 2) showed many lymphoplasma cell infiltrates, a few foreign body-type giant cells, some lymphoid follicle formation, a few eosinophil foci, and fibrosis with a few background foci comprising large numbers of neutrophils. Several plasma cells were immunohistochemically positive for CD138, immunoglobulin G (IgG), and IgG4. Analysis of plasma cells revealed more than 50 IgG4-positive cells/high-power field and an IgG4/IgG-positive plasma cell ratio >40%. These results indicated acute and chronic inflammation. Further evaluation is recommended to rule out IgG4-RD.

The patient was transferred to the Rheumatology Department. Laboratory data, including a complete blood count and blood chemistry, were unremarkable. The erythrocyte sedimentation rate was normal and the C-reactive protein level was 0.31 mg/dL (0–0.3). The results of serologic tests were as follows: serum IgG, 1462 mg/dL (700–1600); IgA, 190.8 mg/dL (70–400); IgM, 106.6 mg/dL (40–230); IgE, 511 IU/mL (0–100 IU/mL). A fluorescent antinuclear antibody test (1:40) was negative, and the rheumatoid arthritis (RA) factor levels were within the normal range. The serum IgG4 level was 69.2 mg/dL (3.9–86.4). Low-dose chest computed tomography and abdominal computed tomography scans were performed. The findings were unremarkable, except for a hepatic cyst. Despite a serum IgG level within the normal range and no involvement of other organs associated with IgG4-RD, we diagnosed the patient with probable IgG4-RD based on comprehensive IgG4-RD diagnostic criteria.^[9]^ Since IgG4-RD generally responds well to glucocorticoid therapy, we started prednisolone treatment. After 1 week of follow-up, the patient soft tissue swelling improved, and after 4 months of follow-up, it completely disappeared.

### 2.2. Case report 2

An 87-year-old woman presented with a palpable soft tissue mass in the right fifth metacarpophalangeal joint (MPJ) of the right hand that had persisted for 2 months. The mass was painful and rubbery, but there were no local signs of infection or constitutional symptoms, such as fever, weight loss, or malaise. The patient had no history of trauma to the area. The patient had a history of diabetes, hypertension, hypertriglyceridemia, and unstable angina.

**Figure 3.**
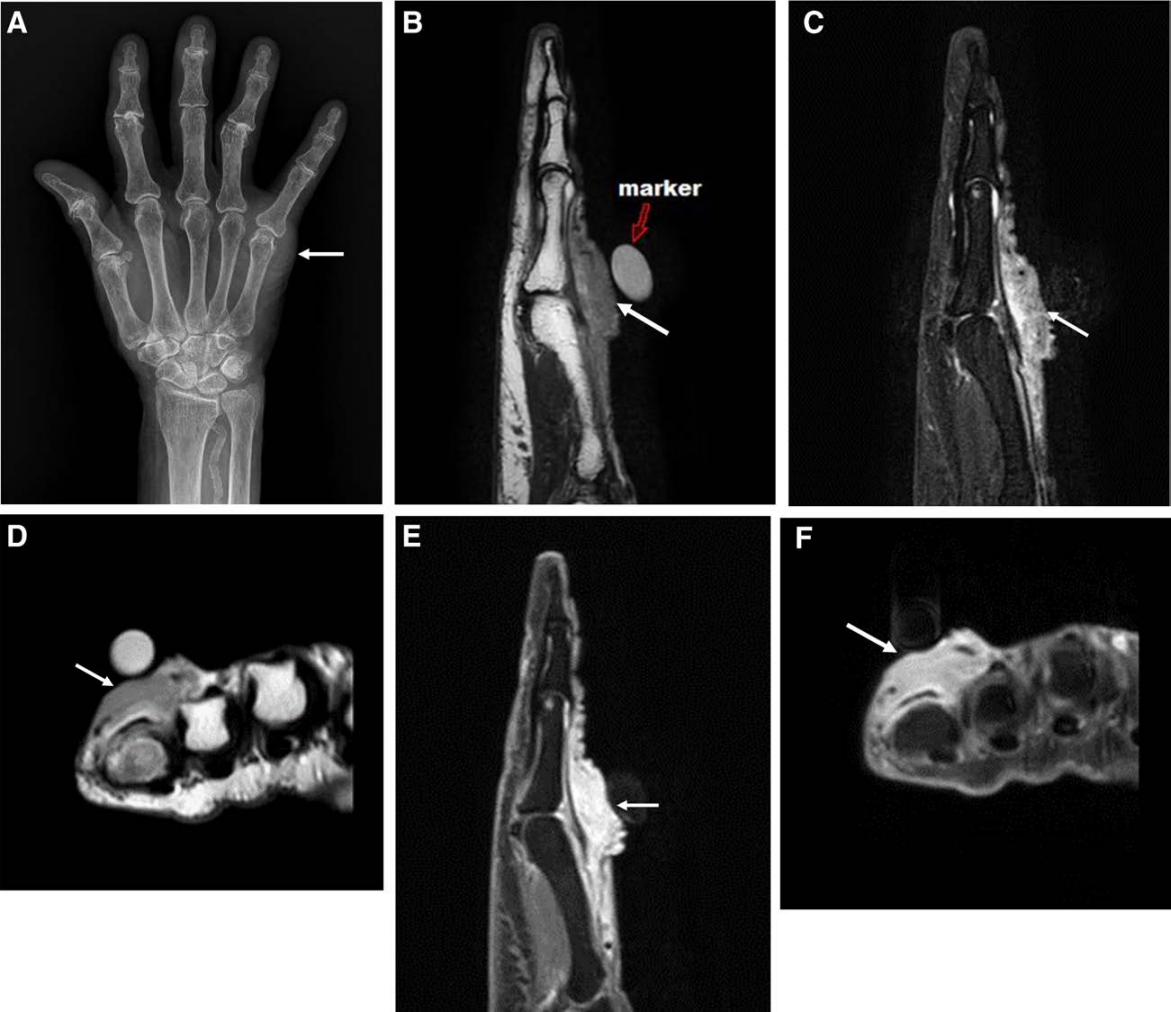
Plain anteroposterior radiograph of the hand shows focal soft tissue swelling (arrow) around metacarpal joint (MPJ) of the little finger (A). MRI of the hand demonstrates an ill defined soft tissue mass (arrows) with low T1 SI (B and D) and intermediate T2 SI (C) in the dorsal subcutaneous aspect of MPJ level in the little finger. Postcontrast fat suppressed T1WI (E and F) shows well, heterogeneously enhancement of the mass (arrows) with neurovascular infiltrative appearance. MRI = magnetic resonance imaging.

Plain radiography of the right hand revealed degenerative osteoarthritis with joint space narrowing in the proximal and distal interphalangeal joints of all fingers and focal soft tissue swelling in the MPJ of the little finger. Vascular calcification was also observed in the distal forearm (Fig. 3A).

The patient underwent MRI of her right hand (Fig. 3B–F). A 2 cm sized, ill-defined mass with low T1 and intermediate T2 SI was observed in the fifth finger MPJ. It was located in the subcutaneous and cutaneous tissues of the dorsal aspect of the extensor digitorum tendon. The mass was internally heterogeneous with cutaneous enhancement and infiltration into the superficial neurovascular bundles. Based on these MRI findings, a malignant tumor (such as UPS, fibrosarcoma, or dermatofibrosarcoma protuberans) seemed more likely than a pseudotumor or an inflammatory mass.

An orthopedic surgeon performed an incisional biopsy. The soft-tissue mass was located in the deep layer of the dorsum of the right fourth and fifth MPJ. The mass was an ill-defined, firm nodule.

**Figure 4.**
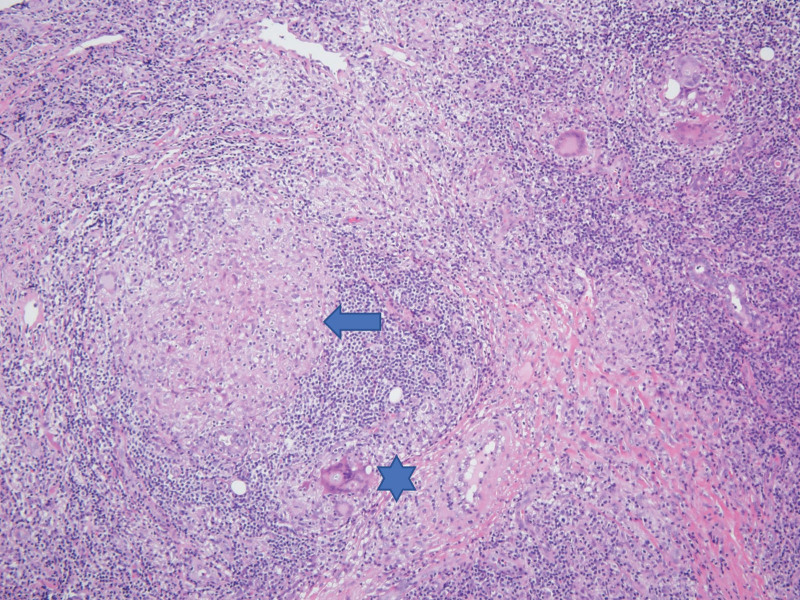
Chronic granulomatous inflammation (arrow) consists of collection of epithelioid cells, foreign-body type giant cells (star), lymphocytes, and plasma cells.

Pathological examination showed collections of epithelioid cells, foreign body type multinuclear giant cells, lymphocytes, and plasma cells (Fig. 4). There was no evidence of necrosis. Polymerase chain reaction for tuberculosis was negative. Therefore, chronic granulomatous inflammation was diagnosed.

The patient was started on nonsteroidal anti-inflammatory drugs. After 2 weeks of follow-up, the patient soft tissue swelling subsided. After 7 months of follow-up, no recurrence was observed.

## 3. Discussion

Tumor-like lesions presenting as masses in the soft tissues of the hand and wrist can be mistaken for more serious conditions.^[8]^ Imaging findings for pseudotumorous lesions are similar to those for true soft tissue tumors; therefore, it is important to conduct a comprehensive differential diagnosis, as an accurate diagnosis is essential for the selection of appropriate medical and surgical treatments. A correct diagnosis can often be made through combined consideration of the patient medical history and findings from physical examination, plain radiography, ultrasound, and MRI. Biopsies should be considered only in cases in which the diagnosis remains uncertain.^[5,8]^ Bilfeld et al described a 3-step diagnostic strategy for assessing masses in the hand and wrist, ruling out pseudotumors as the first step. They described the etiological analyses based on the anatomical location of the mass. Pseudotumors can manifest as synovial, muscular, osseous, vascular, or subcutaneous lesions.^[6]^

There are numerous possible diagnoses of tumorlike lesions in the soft tissue. These include anatomical variants such as accessory muscles; cystic lesions such as synovial, ganglion, and tendon sheath cysts; post-traumatic lesions such as myositis ossificans and foreign body granulomas; epidermoid cysts; infectious and inflammatory lesions such as rheumatoid nodules; annular granulomas; crystal diseases such as gout and pseudogout; and amyloidosis.^[5,8]^ In a study of 134 patients with hand and wrist swelling, the most common cause was ganglions (26.86%), followed by non-tumor tendon pathology (23.13%), articular diseases (5.97%), and anatomical variants (4.47%). Soft-tissue tumors were found in 25% of patients. These were predominantly benign, with only 3 malignant cases.^[7]^ In a study of indeterminate soft tissue masses of the hand and wrist by Sookur et al, non-neoplastic lesions accounted for only 4 of 39 cases (10%). These include nodular fasciitis, myositis ossificans, granulomatous necrotizing inflammation, and an inflammatory fibrous mass.^[10]^ Soft tissue pseudomasses in the hand can be caused by inflammatory pseudotumors, gout, or RA tenosynovitis. Inflammatory pseudotumors are thought to be an abnormal inflammatory response to infection or trauma; however, their occurrence in the hands and wrists is rare.^[11]^

Tumors of the hands and wrists are diverse but mostly benign. Soft tissue sarcomas in this location are rare, and MRI is required for tumor characterization. MRI findings suggestive of malignancy include poorly defined margins, neurovascular invasion, peritumoral edema, inhomogeneous signals, and intense heterogeneous enhancement.^[1,6]^

MRI is widely known to have a limited capacity to distinguish between benign and malignant soft tissue tumors.^[1,3,7,11–13]^ However, specific diagnoses can be made for soft tissue tumors of the hand and wrist owing to the high prevalence of benign lesions in this area and characteristic MRI findings. Benign soft-tissue masses that exhibit distinct MRI features include lipomas, ganglia, giant cell tumors of the tendon sheath, arteriovenous malformations, and fibrolipomatous hamartomas.^[3,7]^ In the absence of distinctive findings, an accurate diagnosis becomes challenging, and malignancy cannot be ruled out.

In both cases, the mass had ill-defined margins, perilesional edema, and heterogeneous imaging signals with intense enhancement and appeared to be infiltrating neurovascular structures. Therefore, our MRI results raised suspicion of malignancy. Both patients underwent incisional biopsy, and their final diagnoses were IgG4-RD-induced inflammation in the first case and chronic granulomatous inflammation in the second case.

IgG4-RD is a systemic, immune-mediated disease that can cause fibroinflammatory lesions and affect multiple organs and tissues. Elevated serum IgG4 concentrations are often, but not always, observed in patients with IgG4-RD. The disease is characterized by tumor-like swelling of the involved organs and prominent lymphoplasmacytic infiltration, including dense fibrotic IgG4-positive plasma cells arranged in a storiform pattern.^[9,14–19]^ Pathological examination is the gold standard for diagnosis.

IgG4-RD can affect numerous organs, with the pancreas being the most common organ. Other organs implicated in IgG4RD are the lung, bile duct, kidney, prostate, retroperitoneum, pituitary gland, submandibular gland, lacrimal gland, and thyroid gland.^[9,14–19]^

Umehara et al proposed the 2020 revised comprehensive diagnostic criteria for IgG4-RD: involvement of one or more organs, as shown by swelling, mass, or nodules defined clinically or radiographically; Serum IgG4 levels >135 mg/dL; and positivity for 2 of the following 3 criteria: dense lymphocyte and plasma cell infiltration with fibrosis, more than 10 IgG4-positive cells/high-power field, an IgG4-positive/IgG-positive cell ratio >40% on histopathological examination, and storiform fibrosis or obliterative phlebitis. A definite diagnosis of IgG4-RD can be made if all the 3 criteria are met. A diagnosis of probable IgG4-RD can be made if the first and third criteria are met and possible IgG4-RD if the first and second criteria are met^.[9]^

Based on these criteria, our first case was diagnosed as a probable IgG4-RD soft tissue pseudotumor because despite the lack of elevation in serum IgG4 levels, there was a high rate of IgG4-positive cells in the tissue.

Hamano et al have demonstrated that, as in our case, the pathological findings from a patient biopsy specimen may reveal abundant IgG4-positive plasma cells, even in cases with normal serum IgG4 levels, which can aid in accurate diagnoses.^[17]^

Musculoskeletal mass formation due to biopsy-proven IgG4-RD is rare. Only 1 case of IgG4-RD as a soft tissue mass has been reported in the literature.^[19]^ The mass was seen in the thigh of an adolescent patient and showed an infiltrative growth pattern. It extends along the neurovascular bundle in the left iliopsoas muscle and intermuscular space. However, the first case presented herein is the first to report an IgG4-RD soft-tissue mass in a wrist-mimicking malignancy.

Infiltrating soft tissue masses of the wrist and hand have a broad spectrum of causes, both benign and malignant.^[12]^ In our study, both patients presented with some features of malignancy. In the first case, neurovascular infiltration and adjacent fascial enhancement were observed; in the second case, cutaneous enhancement was seen. Differential diagnosis of such masses considers primary superficial soft tissue sarcoma as the main possibility, but may also include inflammatory pseudotumors, myofibroblastic tumors, localized inflammatory pathology, tuberculosis, fungal infections, RA, and sarcoidosis.^[19–21]^

Sarcomas have variable appearances, but most manifest as large aggressive masses, with heterogeneous SI on T1 and T2WI owing to areas of necrosis, hemorrhage, and calcification.^[19]^ Synovial sarcomas are slow-growing, and calcification is observed in 30% of these lesions. Features may include hemorrhage, fluid-fluid levels, and the “triple sign,” which are areas of high, intermediate, and low SI on T2WI due to the combination of cystic elements (hemorrhage and necrosis) and solid elements. Epithelioid sarcomas are the most common soft tissue malignancies of the hand in young adults, with approximately 60% of cases involving the flexor surfaces of the finger, hand, and wrist. These sarcomas tend to propagate along the fascial planes, nerves, tendons, and areas of chronic inflammation around the tumor periphery, sometimes mimicking infections. Fibroblastic myxoinflammatory sarcomas are low-grade subcutaneous tissue tumors that commonly affect the hands and feet. The myxoid component shows enhancement, and its border may be blurred with comet tail aponeurotic extensions.^[2]^ UPS affects the subcutaneous tissue and is most common in older patients. Dermatofibrosarcoma protuberans are typically soft tissue masses of the skin and subcutaneous adipose tissue with linear extensions along the skin surface. The appearance of myofibroblastic tumors on imaging varies from ill-defined infiltrating lesions to well-circumscribed soft tissue masses containing differing proportions of inflammatory and fibrotic components.^[20]^ A hypointense band or foci on T2WI MRI is generally the fibrous component of the tumor. A linear fascial extension to a soft-tissue mass is believed to be indicative of the infiltrative growth pattern of a tumor and is a common MRI feature of myofibroblastic tumors and malignancies such as myxofibrosarcoma and UPS.^[21]^

The analyses of our cases revealed tumorous masses that appeared infiltrative with apparent neurovascular invasion. However, on contrast-enhanced MRI, the neurovascular structures in the enhanced lesions appeared to be intact. This suggests that inflammation is more likely to occur than sarcoma.

Before hand and wrist surgery, soft tissue masses must be classified as determinate or indeterminate lesions. Determinant masses are benign and can be accurately diagnosed using MRI. Indeterminate masses require biopsy for an accurate diagnosis. Indeterminate masses may be either benign or malignant. However, malignant masses can be mistaken for determinate lesions and benign masses can be classified as indeterminate lesions, as in the 2 cases presented in this paper. For indeterminate soft tissue masses with a high probability of malignancy, incisional or imaging-guided biopsies are required.^[22,23]^ Thus, as our patients’ lesions were classified as indeterminate, incisional biopsies were performed to allow for accurate diagnoses.

In conclusion, the 2 cases presented here are rare instances of pseudotumors of the hand and wrist that mimicked malignancy on MRI. Our first case was a rare IgG4-RD inflammatory pseudotumor of the wrist. The second case presented with granulomatous hand inflammation with an ill-defined infiltrative appearance of a malignant tumor.

Most benign soft tissue masses of the wrist and hand have distinctive MRI features. If a lesion cannot be confidently characterized as a benign entity, it is deemed indeterminate, and a biopsy and histologic analysis should be performed to ensure accurate diagnosis and appropriate treatment.

## Author contributions

**Conceptualization:** Yeon Soo Lee.

**Data curation:** Sangeun Park.

**Supervision:** Yeon Soo Lee.

**Writing – original draft:** Jong Ok Kim.

**Writing – review & editing:** Jong Ok Kim, Yeon Soo Lee, Sangeun Park.
